# Biochar effects on phosphorus availability in agricultural soils: A meta-analysis

**DOI:** 10.1038/s41598-019-45693-z

**Published:** 2019-06-27

**Authors:** Bruno Glaser, Verena-Isabell Lehr

**Affiliations:** Martin Luther University Halle-Wittenberg, Institute of Agronomy and Nutritional Sciences, Soil Biogeochemistry, von-Seckendorff-Platz 3, 06120 Halle/Saale, Germany

**Keywords:** Element cycles, Geochemistry

## Abstract

Phosphorus (P) is a limiting nutrient for plants and an essential element for all life on Earth. As the resources of phosphate rock are depleting, new management tools for environmentally friendly P fertilizers are needed. In order to achieve this, recent studies have proposed to use biochar, a carbon-rich solid product of thermochemical conversion of biomass with minimal or zero oxygen supply, as slow-release P fertilizer. However, the effects of biochar on plant-available P in soils have been reported to be variable. Therefore, we quantitatively evaluated existing peer-reviewed data using meta-analysis to draw general conclusions. In the present study, we evaluated 108 pairwise comparisons to their response of biochar application on P availability in soils. Our results indicate that biochar can act as a short-, mid-, and long-term P fertilizer with its effect depending on feedstock, pyrolysis temperature and application amount. Overall, the addition of biochar significantly increased the P availability in agricultural soil by a factor of 4.6 (95% confidence interval 3.4–5.9), independent of the used feedstock for biochar production. Only biochar application amounts above 10 Mg ha^−1^ and biochar produced at temperatures lower than 600 °C significantly increased the P availability of agricultural soils. The application of biochar to acid (pH < 6.5) and neutral soils (pH 6.5–7.5) significantly increased plant-P availability by a factor of 5.1 and 2.4, respectively (95% confidence interval 3.5–6.7 and 1.4–3.4, respectively), while there was no significant effect in alkaline soils (pH > 7.5). Taken together, this meta-analysis shows that biochar significantly enhances plant-available P in biochar-amended soils at least for five years.

## Introduction

Phosphorus (P) is a major nutrient, essential for plant growth that cannot be substituted by any other element^1^. Phosphorus is mainly derived from non-renewable phosphate rock^2^ and its application as inorganic P fertilizers increased rapidly since 1950^3^ due to higher food demand for a growing world population^4^. Phosphate rock is mined faster than it is deposited^1^ and it is estimated that the reserves of phosphate rock, based on current production rates of P fertilizers, will only last for the next 300–400 years^5^. However, P is not only supplied to soil as mineral fertilizer but also in form of agricultural residues, e.g. animal manure or sewage sludge.

Due to the fact that in common agricultural practice more P is being applied than required by crops^6,7^, P accumulates in soils and causes eutrophication of water bodies mostly via surface runoff^8,9^. Annual P fluxes from soil to ocean are estimated at 4–6 Pg per year being about twice as much as natural P fluxes in the past^10^. Especially in Northern Europe, P loss from agricultural soils is a major concern due to the accelerated eutrophication of the Baltic Sea and many inland surface waters. Another environmental risk caused by the use of P fertilizer is the accumulation of radioactive contaminant in soil and food chain as phosphate rock can contain high concentrations of uranium^11^. Although P is accumulated in soils through excessive use of fertilizers, a major global problem is the actual availability for plants of soluble phosphorus in agricultural soils^12^. Phosphorus is highly immobile due to adsorption, precipitation and conversion into the organic form^13^. Phosphorus has a very low solubility and is only available for plant uptake in its inorganic form as HPO_4_^2−^ or H_2_PO_4_^−^ and H_3_PO_4_^14^.

Facing the outlined problems, new techniques must be developed to ensure the environmentally friendly recycling of P^7^. An efficient P use and an intelligent management of the P cycle is essential for achieving a sustainable use of P^2^. Thus, it is important to recycle P from P-rich residues such as manure and sewage sludge to meet the rising need of P for food production^8^.

In order to achieve this, recent studies have proposed to use biochar, a carbon-rich solid product of thermochemical conversion of biomass residues with minimal or zero oxygen supply^15^, as slow-release P fertilizer^16–21^. Biochars are produced from a wide range of organic feedstocks^22^ under different thermochemical conditions resulting in a huge variety of properties such as pH value, elemental composition, degree of aromaticity, content of functional groups etc^23^. Thus, the composition of biochars is very heterogeneous^24^ as the surface can show acidic, basic, hydrophobic and hydrophilic properties^25^. It has been reported that biochar can increase the cation exchange capacity (CEC) in soils^26–28^, change soil pH^29,30^ and influences plant access to soil P^31^.

As mineral P resources are decreasing, biochar can play an important role to recycle P from agricultural residues such as manure^32^ or sewage sludge^33^. Through thermochemical treatment of manure, P can be fully recovered and enriched in the product, which shows the same fertilization efficiency as mineral fertilizers^18^. Enrichment of P is achieved by a concentration effect as P volatilize at temperatures above 700 °C, compared to carbon (C), which starts to volatilize at 100 °C^34^.

Because biochar properties can differ widely, it is important to examine which characteristics of biochar and its production processes have an influence on P availability in agricultural soils. As a large number of studies quantify the effect of biochar on plant-available P in soils, meta-analysis is a helpful tool to summarize the results of each study in order to reach general conclusions. In the present meta-analysis, we investigated the effect of biochar on plant-available P in agricultural soils. In order to further evaluate key properties of biochar in agriculture soils, the effects of biochar feedstock type, application amount, pyrolysis temperature, soil pH, and duration of the experiment have been investigated.

## Material and Methods

### Source of data

A systematic literature search of published articles and book chapters was performed by using ISI Web of Science. Key words used for the search were: phosph* AND avail* AND *biochar* (title) during the period 1980 to 2016. ISI Web of Science yielded 194 results. We excluded studies, which did not include sufficient information about the effect of biochar on phosphate availability in agricultural soils or being not relevant for this meta-analysis. We selected literature by using the following criteria: (i) studies with a control measurement of un-amended soil, (ii) studies using the same P extraction methods for control and amended soil, (iii) no additional P fertilizer was applied, (iv) studies incorporating biochar into the soil; if biochar was only spread on the surface, the study was excluded; (v) biochar produced by pyrolysis; char produced by wildfire was excluded; (v) only agricultural soils.

If measurements were taken at multiple points of time, only the last measurement was included. If data was only presented in figures, GraphClick Version 3.0.3 was used for data extraction.

Besides the plant-available soil P content, further information and data was extracted from each study including: (i) area (region, latitude, climate); (ii) soil properties (soil type, texture, depth, pH, CEC, available P extraction method; (iii) experimental setup (type of experiment, duration of experiment); (iv) biochar characteristics (production method & temperature, carbonization time and feedstock, pH, application amount, CEC and available P). If essential data was not available in the study itself, the corresponding authors were contacted for providing the necessary information. Studies were excluded, if the necessary information could not be obtained.

### Data categorization

In order to better understand the response of biochar on P availability in agricultural soil, the results of the studies were grouped into biochar properties including feedstock type, application amount, pyrolysis temperature, and experimental duration.

### Meta-analysis

The effect of biochar application on P availability in agricultural soils was calculated using the response ratio (R), which is the mean of the biochar-treated soil divided by the mean of the control group without biochar. Natural log transformation of the response ratio is useful for obtaining more appropriate statistical properties (e.g. symmetric distribution) because ratios in general have poor statistical properties^35^. Thus, the natural log of the response ratio ln(R) was calculated by using the following formula^36^:

$$\mathrm{ln}({\rm{R}})=ln(\frac{{X}_{E}}{{X}_{C}})$$

where *X*_*E*_ is the mean plant-available phosphate content of the soil amended with biochar and *X*_*C*_ the mean plant-available phosphate content of the un-amended soil of individual treatments.

Statistical analyses were performed using R 3.0.3. Normal distribution of data was tested using histograms and Shapiro-Wilk Normality Test. Data were not normally distributed, therefore, they were log-transformed. The log-transformed data showed a right-skewed distribution and differed significantly from a normal distribution (W = 0.90046, p-value = 0.0000007). Outliers were visually identified by using Box-and-Whiskers-Plots. Subsequently, a nonparametric outlier test by Walsh (1958) was performed for excluding outliers of ln(R) at α = 0.10. The Walsh test indicated that two values were outliers and those were removed from the data set.

Owing to the fact that many studies did not include the measure of variance, an un-weighted meta-analysis was performed. Thus, also studies that did not provide explicit statements of variance could be included. This allowed a higher number of studies to be included in this meta-analysis. In addition, an un-weighted meta-analysis allows the more variable and more realistic field studies to influence conclusions with the same impact as the less variant and higher replicated laboratory and greenhouse studies^37,38^. Therefore, weighted meta-analysis can under- or overestimate response ratios.

In order to better understand impacts of biochar addition on P availability in agricultural soil, a regression analysis of the log-transformed response ratio was conducted to examine whether temperature and application amount significantly correlated with ln(R). For this analysis, the original data (more precisely: the exact values of the pyrolysis temperature or the biochar application amount) and not the grouped data was used. In case a range of temperature or a range of the application amount was given, the average value was taken. Additionally, the feedstock groups were added to the graphs. An ln(R) value equal to 0 indicates that no change in soil P availability could be obtained through biochar addition.

For the purpose of interpretation and presenting the results in graphs, the natural log-transformed data was transformed from ln(R) into R, thus *R* = *e*^*ln(R)*^. A 95% Confidence Interval (CI) of the overall mean response ratio and the mean response ratio of each group was calculated using the following formula for calculating the upper and lower limit:

$$\begin{array}{c}{{\rm{Cl}}}_{{\rm{upper}}}:\bar{R}+1.96\ast \frac{\sigma }{\sqrt{n}}\\ {{\rm{Cl}}}_{{\rm{lower}}}:\bar{R}-1.96\ast \frac{\sigma }{\sqrt{n}}\end{array}$$

with $\bar{R}$ denoting the back-transformed mean response ratio, 1.96 the confidence coefficient, σ the standard deviation and n the number of individual treatments.

An R equal of 1 indicates that biochar treatment had no effect on P availability in the amended soil. A response ratio below 1 indicates a lower plant-available P content and an R above 1 indicates a higher plant-available P content in the tested soils. Most figures were outlined as forest plots showing the mean ratio of biochar-amended soils vs control without biochar as dot and the 95% confidence interval as bar for each group. Following^39^, a mean response ratio of each group was considered to be significantly different at p < 0.05 from each other if the 95% confidence intervals were not-overlapping. Means were considered significantly different from 1 at p < 0.05 if the 95% CI were not overlapping 1. The overall mean response ratio was presented in each group and varied among the categorical group, depending on the number of pairwise comparisons included.

## Results

**Figure 1:**
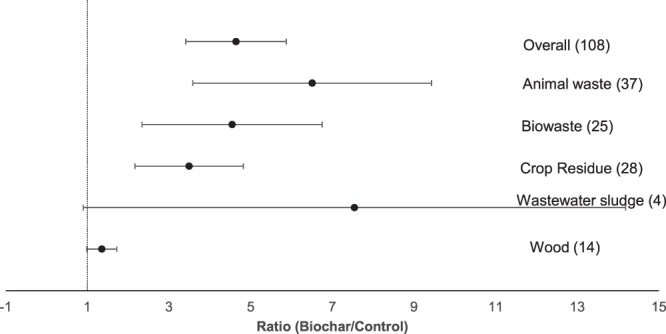
Forest plot showing the influence of different feedstock utilized for biochar production on changes of P availability in agricultural soils. Points represent mean response ratios and error bars show the 95% confidence interval. The dashed line was drawn at response ratio = 1. Number in parenthesis show the number of pairwise comparisons on which the statistic is based. For the explanation of feedstock categories see section 2.2.

In total, 25 published articles (including a dataset of 108 pairwise comparisons), were included in this meta-analysis (for original data see Supplementary Table 1). These studies comprised biochar experiments from all over the World, including Europe (30.3%), North America (27.3%), Asia (24.2%), Africa (9.1%) and Australia (9.1%). Most experiments were carried out as incubation studies (80%), followed by pot studies with 13% and only 7% of the experiments were designed as field studies. In the following, the results of each group are presented. In general, the addition of biochar significantly increased the P availability in agricultural soil by 460% (Fig. 1).

### Biochar feedstock

In Fig. 1, the effect of the different biochar feedstock on P availability is presented. The highest and lowest effect on P availability in agricultural soils were obtained by biochars produced from wastewater sludge and wood, both being not significant (Fig. 1). All other biochars showed a significantly positive response on P availability in agricultural soils, decreasing in the following order: animal waste > biowaste > crop residues (Fig. 1). However, there are no significant differences between the different feedstock present in the data (Fig. 1).

### Biochar application amount

**Figure 2:**
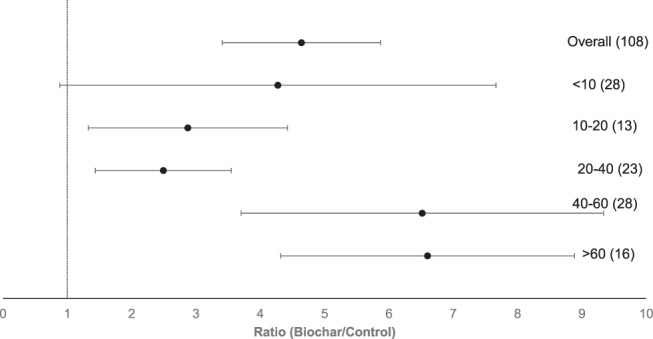
Forest plot showing the influence of different biochar application amount in Mg ha^−1^ on changes of P availability in agricultural soils. Points represent mean response ratios and error bars show the 95% confidence interval. The dashed line was drawn at response ratio = 1. Number in parenthesis show the number of pairwise comparisons on which the statistic is based.

**Figure 3:**
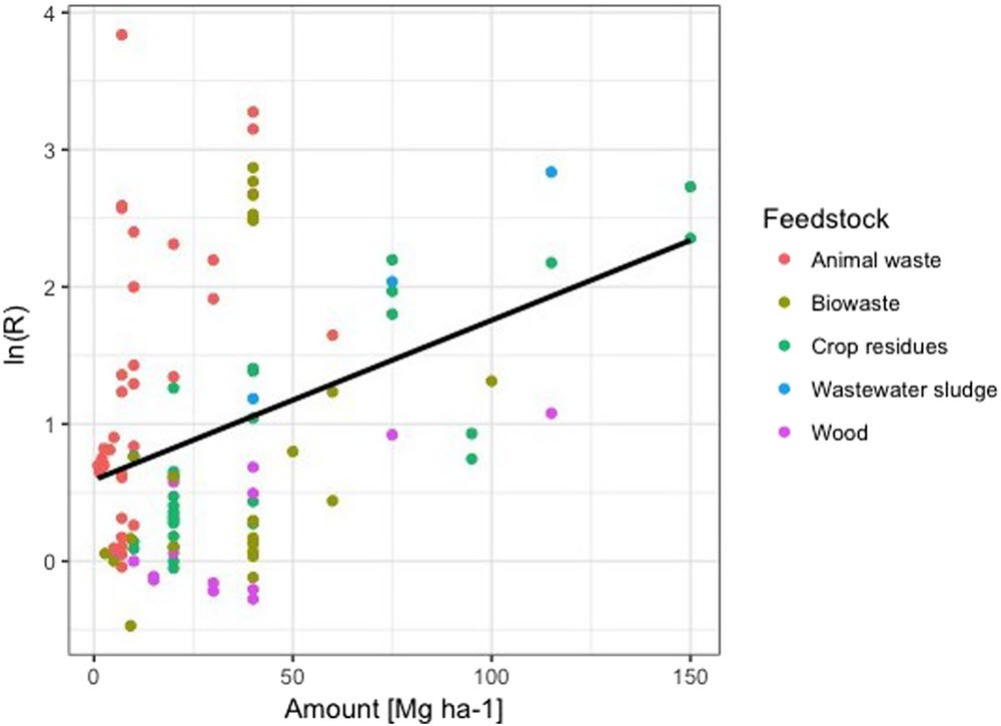
Log-transformed response ratio (biochar/control) of P availability in soils amended with biochar as a function of biochar application amount in Mg per ha (r^2^ = 0.14, p < 0,001). Each symbol represents one study; colours represent feedstock group.

The effect of the amount of biochar added to soil on P availability is presented in Fig. 2. Low application amounts (less than 10 Mg ha^−1^) showed a non-significant response. Biochar application amounts between 10 and 20 Mg ha^−1^ did not significantly differ from the other application amounts, while P availability at 20 and 40 Mg biochar ha^−1^ was significantly lower than at 40 and 60 Mg biochar ha^−1^ and above 60 Mg biochar ha^−1^ (Fig. 2). Higher biochar application amounts (above 10 Mg ha^−1^) significantly increased the P availability of agricultural soils. As shown in Fig. 3, higher application amounts significantly correlated with the log-transformed response ratio of the P availability (r^2^ = 0.14, p < 0.001).

### Pyrolysis temperature

**Figure 4:**
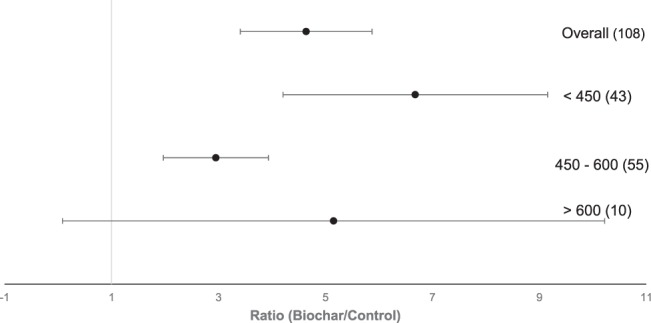
Forest plot showing the influence of pyrolysis temperature of biochar in °C on changes of P availability in agricultural soils. Points represent mean response ratios and error bars show the 95% confidence interval. The dashed line was drawn at response ratio = 1. Number in parenthesis show the number of pairwise comparisons on which the statistic is based.

**Figure 5:**
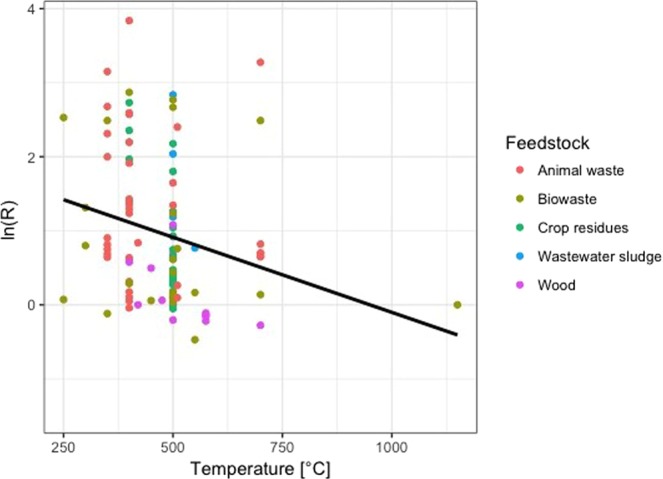
Log-transformed response ratios (biochar/control) of P availability in soils amended with biochar as a function of pyrolysis temperature (r^2^ = 0.06, p ≤ 0.01). Each symbol represents one study; a colour represents a feedstock group.

The influence of pyrolysis temperature on biochar properties in terms of P availability is plotted in Fig. 4. Biochars produced at temperatures higher than 600 °C had no significant effect on P availability. Low temperature biochars (450 °C) and mid temperature biochars (450–600 °C) significantly enhanced the P availability in soils amended with biochar and also significantly differed from each other. As shown in Fig. 5, the log-transformed R of P availability significantly declined with increasing temperature of biochars (r^2^ = 0.06, p < 0.01).

### Soil pH

**Figure 6:**
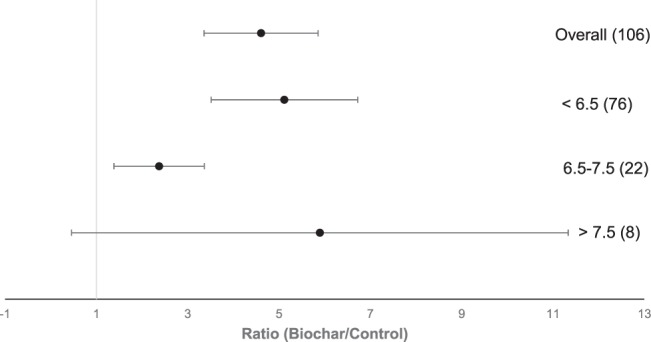
Forest plot showing the influence of biochar on changes of P availability in agricultural soils regarding the pH of soils. Points represent mean response ratios and error bars show the 95% confidence interval. The dashed line was drawn at response ratio = 1. Number in parenthesis show the number of pairwise comparisons on which the statistic is based.

The application of biochar to acid soils (pH < 6.5) had the strongest positive effect on P availability (Fig. 6). The effect is significantly higher than in neutral soils (pH 6.5–7.5). No significant response was observed in alkaline (pH > 7.5) soils (Fig. 6).

### Duration of the experiment

**Figure 7:**
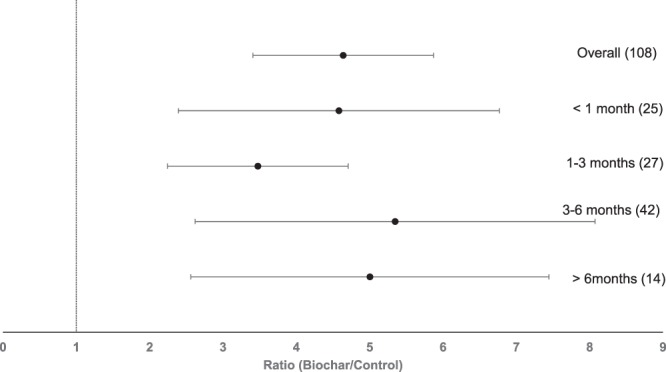
Forest plot showing the influence of the duration of experiments with biochar on changes of P availability in agricultural soils. Points represent mean response ratios and error bars show the 95% confidence interval. The dashed line was drawn at response ratio = 1. Number in parenthesis show the number of pairwise comparisons on which the statistic is based.

There was no significant effect of the experiment duration on P availability (Fig. 7). While all groups had a significant response on the P availability in agricultural soils, individual groups did not significantly differ from each other (Fig. 7).

## Discussion

On average, in this meta-analysis of 108 pairwise comparisons, the application of biochar significantly increased the plant-available P content in agricultural soil despite variability in feedstock, climate, soil type or production methods of biochar. The overall mean response of biochar to plant-available P was significantly positive. Thus, biochar appears to be a helpful tool for recycling P in agricultural systems.

Biochars derived from animal residues, agricultural residues and crop residues show a positive response to plant-available P in biochar-treated soils. The number of studies on wastewater sludge was considerably lower (only four pairwise comparisons) and, therefore, the results are highly variable, which is likely reflected in its large variation and possibly allows poor determination of significant effects. To define the influence of wastewater sludge-derived biochar on the P availability in soils, more studies should be carried out. The group animal residues comprising different types of manure and animal bone as biochar feedstock had a significant positive response to P availability. Since the total amounts of P in annually produced livestock manures are higher than the world production of P fertilizers^40^, thermochemical conversion of manure into biochar seems a helpful tool to minimize the production of mineral P fertilizer. Recycling P from organic residues has environmental benefits compared to direct land application (e.g. protection of water bodies) and can provide a continuous P source for soils^18,32,41^. However, P solubility in charred biomass is reduced compared to uncharred material^42^ due to bonding of P with multivalent metal cations in biochar^32^. Thus, production of biochar alters P form in agricultural residues and can change the environmental fate of P^32^.

For wood-derived biochar, no effect on P bioavailability could be observed and additionally, the level of variety for wood biochar is very small. Therefore, it seems not to be the ideal candidate to be considered as a P fertilizer, but may have other benefits. In addition, this indicates that biochar does not liberate P from soil, at least not for wood-derived biochar.

It can be concluded that biochars produced from P-rich feedstock provide higher amounts of plant-available P compared to P-poor feedstock, probably providing P from biochar itself. In addition, wood-derived biochar does not enhance plant-available P in soil. For all other biochars, this aspect cannot be evaluated in this study, as the response ratio does not allow do differentiate between P derived from biochar and from soil.

There is a weak but significant correlation between application amount and plant-available P in biochar-treated soil. Plant-available P positively responded to biochar application above 10 Mg ha^−1^. However, for defining thresholds of application amounts, not only the increase of nutrient supply in response to biochar addition should be considered but also the potential effect on increasing nutrient leaching. It has been demonstrated that P leaching from biochar-amended soils has a potential for water pollution^43,44^. In contrast to these results it has been reported that biochar has the ability to adsorb orthophosphate as well as organic P compounds and thus reduce the leaching of P^45^. Clearly, there is still no accurate statement possible regarding the effect of biochar on leaching of P from soil in this study. However, the environmental fate of P depends upon its solubility in soils^32^. Recommendation of optimum application amounts must also consider the response of application amount to plant growth since negative effects on plant growth through biochar addition have been reported. Biomass has been reported to decrease at application amounts above 60 Mg ha^−1^ ^46^ and, above 120 Mg ha^−1^ ^47,48^.

Biochars produced at temperature below 450 °C caused a statistically significant increase of plant-available P in amended soils. In addition, with increasing pyrolysis temperature the effect of biochar on P availability decreases significantly. Comparable results can also be observed among different studies, where the extractable P declined from high to low temperature biochars^31,49^. It has been observed that, due to rising temperatures, organic P species disappear in favour of inorganic P compounds^21,50^. P starts to volatilize at temperature above 700 °C, therefore the P content of the feedstock can be recovered in low- and mid-temperatures biochars, but not in high-temperatures biochars^17^. Compared to P, carbon (C) present in the biochar already volatilize at 100 °C^34^ causing an enhancement of P and cleaving of organic P bonds. As a result, biochars contain soluble P salts, which are associated with the charred material. Thus, the present results and research on this topic suggest that biochars produced below 600 °C can have positive effects on P plant availability.

Biochar incorporation into soils was most effective on P availability in acidic soils. The results show that there was no significant effect on plant-available P in alkaline soils. The P solubility in alkaline soils is mainly regulated by the interaction of P with Ca^2+^, whereas in acidic soils the plant availability of P is mainly regulated by Al^3+^ and Fe^2+^/Fe^3+^ by forming Al-and Fe-phosphates. As soon as the application of biochar to acid soils increases soil pH, the P sorption onto Fe and Al oxides decreases^51^. Moreover, biochar itself can be a source of Ca and Mg and those elements can have an effect on P availability^52,53^. A high content of Ca and Mg in biochar and/or the presence of calcareous substances can cause the formation of calcium and magnesium phosphate which can reduce plant uptake of P^51^. On the other hand, studies hypothesizing that through the application of biochar to acidic soils phosphate bonded with free cations such as Fe^3+^, Al^3+^, Ca^2+^ and Mg^2+^ dissolved and released plant-available P^54^. In a two years field study in calcareous soil, no effect on P availability could be observed by biochar application^55^.

In this meta-analysis of 54 different biochars, 48 biochars had an alkaline pH. Thus, mainly alkaline biochars were added to acid soils supporting the hypothesis that biochar addition has a liming effect on acidic soils^56^ and, therefore, improves the nutrient use efficiency^57,58^. Consequently, it seems advisable to add alkaline biochars to acidic soils and acid biochars to alkaline soils for enhancing the plant-available P in soils^51^.

Studies included in this meta-analysis ranged from incubation studies of two days up to field studies of five years. No significant change of biochar response over time has been observed. Therefore, it seems that the positive effect of biochar on plant-available P is not limited to a short-term positive effect on soil pH. Given the significant effects that are present in all time period groups it can however, be concluded that biochar application increases P availability in agricultural soil in short-, mid- and long-term time spans. The present results resemble the finding that no explicit change over time in plant-available P could be observed^8^.

## Conclusions

Results from this meta-analysis demonstrated that biochar has the potential to enhance plant-available P in biochar-amended soils and could be a sustainable strategy to complement conventional P fertilizers. Biochar produced from crop, agricultural or animal residues showed the highest effect, as well as biochars produced at temperatures below 450 °C. Biochar should be applied at amounts above 10 Mg ha^−1^ to achieve effects on P availability. Unfortunately, little information is available regarding the P content of biochar and its correlation with plant-available P in soil. This would be an interesting subject to study. Clearly, more research on the effects that application amounts of biochar has on plant growth is necessary in order to avoid negative effects. Nevertheless, for an adequate recommendation and a best practice management, further investigations about the long-term retention-release patterns should be carried out. For instance, the sorption capacity of biochars need to be fully understood in order to describe the P retention in soils. This is important, so that P released from biochar does not become a continuous source to downstream water bodies, adding to water pollution. In addition, following meta-analysis should also examine the effect of treated biochars due to research suggesting that activation^59^, composting^53,60–63^ and joint-application with urea^64^ show positive effects on nutrient availability in biochar-amended soils. Finally, this present meta-analysis suggests that the application of biochar to agriculture soils appears to be a reasonable tool in an increasingly P-finite agricultural industry.

## Supplementary information

Supplementary Dataset 1

## References

[1] Neset TSS, Cordell D (2012). Global phosphorus scarcity: Identifying synergies for a sustainable future. J. Sci. Food Agric..

[2] Cordell, D., Drangert, J. & White, S. The story of phosphorus: Global food security and food for thought. **19**, 292–305 (2009).

[3] Smil V (2000). Phosphorus in the Environment_ Natural Flows and Human Interferences _ Annual Review of Environment and Resources. Annu. Rev. Energy Environ..

[4] Koning NBJ (2008). Long-term global availabil-ity of food: Continued abundance or new scarcity? *Wageningen J*. Life Sci..

[5] Van Kauwenbergh S.J. *World Phosphate Rock Reserves and Resources. IFDC Technical Bulletin 75*. *International Fertilizer Development Center (IFDC), Muscle Shoals, Alabama, USA* (2010).

[6] Gerber P, Opio C, Steinfeld H (2007). Poultry production and the environment-A review. Fao.

[7] Zoboli O, Zessner M, Rechberger H (2016). Supporting phosphorus management in Austria: Potential, priorities and limitations. Sci. Total Environ..

[8] Bennett EM, Carpenter SR, Caraco NF (2001). Human Impact on Erodable Phosphorus and Eutrophication: A Global Perspective. Bioscience.

[9] Siddique MT, Robinson JS (2003). Phosphorus Sorption and Availability in Soils Amended with Animal Manures and Sewage Sludge. J. Environ. Qual..

[10] Filippelli GM (2008). The global phosphorus cycle: Past, present, and future. Elements.

[11] Chen B, Roos P, Borggaard OK, Zhu YG, Jakobsen I (2005). Mycorrhiza and root hairs in barley enhance acquisition of phosphorus and uranium from phosphate rock but mycorrhiza decreases root to shoot uranium transfer. New Phytol..

[12] Meena RS, Meena PD, Yadav GS, Yadav SS (2017). Phosphate Solubilizing Microorganisms, Principles and Application of Microphos Technology. J. Clean. Prod..

[13] Holford I (1997). Soil phosphorous, its measurements and its uptake by plants. Austalian J. Soil Rresearch.

[14] Schachtman DP, Reid RJ, Ayling SM (1998). Phosphorus Uptake by Plants: From Soil to Cell. PLANT Physiol..

[15] Lehmann, J. & Joseph, S. Biochar for environmental management–an introduction. In *Biochar for Environmental Management* 1–18, 10.1016/j.forpol.2009.07.001 (2009).

[16] Zhao L (2016). Copyrolysis of Biomass with Phosphate Fertilizers to Improve Biochar Carbon Retention, Slow Nutrient Release, and Stabilize Heavy Metals in Soil. ACS Sustain. Chem. Eng..

[17] Wang T, Camps-Arbestain M, Hedley M, Bishop P (2012). Predicting phosphorus bioavailability from high-ash biochars. Plant Soil.

[18] Wang Y, Lin Y, Chiu PC, Imhoff PT, Guo M (2015). Phosphorus release behaviors of poultry litter biochar as a soil amendment. Sci. Total Environ..

[19] Slavich PG (2013). Contrasting effects of manure and green waste biochars on the properties of an acidic ferralsol and productivity of a subtropical pasture. Plant Soil.

[20] Liang Y, Cao X, Zhao L, Xu X, Harris W (2014). Phosphorus Release from Dairy Manure, the Manure-Derived Biochar, and Their Amended Soil: Effects of Phosphorus Nature and Soil Property. J. Environ. Qual..

[21] Uchimiya M, Hiradate S, Antal MJ (2015). Dissolved Phosphorus Speciation of Flash Carbonization, Slow Pyrolysis, and Fast Pyrolysis Biochars. ACS Sustain. Chem. Eng..

[22] Farrell M, Rangott G, Krull E (2013). Difficulties in using soil-based methods to assess plant availability of potentially toxic elements in biochars and their feedstocks. J. Hazard. Mater..

[23] Kloss S (2012). Characterization of Slow Pyrolysis Biochars: Effects of Feedstocks and Pyrolysis Temperature on Biochar Properties. J. Environ. Qual..

[24] Schimmelpfennig S, Glaser B (2012). One Step Forward toward Characterization: Some Important Material Properties to Distinguish Biochars. J. Environ. Qual..

[25] Atkinson CJ, Fitzgerald JD, Hipps NA (2010). Potential mechanisms for achieving agricultural benefits from biochar application to temperate soils: a review. Plant Soil.

[26] Liang B (2006). Black carbon increases cation exchange capacity in soils. Soil Sci. Soc. Am. J..

[27] Glaser B, Haumaier L, Guggenberger G, Zech W (2001). The ‘Terra Preta’ phenomenon: A model for sustainable agriculture in the humid tropics. Naturwissenschaften.

[28] Glaser B, Lehmann J, Zech W (2002). Ameliorating physical and chemical properties of highly weathered soils in the tropics with charcoal–a review. Biol. Fertil. Soils.

[29] Chan KY, Van Zwieten L, Meszaros I, Downie A, Joseph S (2007). Agronomic values of greenwaste biochar as a soil amendment. Aust. J. Soil Res..

[30] Glaser, B. *et al*. Potential of Pyrolyzed Organic Matter in Soil Amelioration. *People’s Repub. China Minist. Water Resour. 12th Int. Soil Conserv. Organ. Conf. Beijing*, *China. Minist. Water Resouces Beijing*, (in Press 421–427 2002).

[31] Zwetsloot MJ (2016). Phosphorus availability from bone char in a P-fixing soil influenced by root-mycorrhizae-biochar interactions. Plant Soil.

[32] Dai L (2016). Biochar: a potential route for recycling of phosphorus in agricultural residues. GCB Bioenergy.

[33] Yuan H, Lu T, Wang Y, Chen Y, Lei T (2016). Sewage sludge biochar: Nutrient composition and its effect on the leaching of soil nutrients. Geoderma.

[34] Novak JM (2009). Characterization of designer biochar produced at different temperatures and their effects on a loamy sand. Ann. Environ. Sci..

[35] Koricheva Julia, Gurevitch Jessica, Mengersen Kerrie (2013). Handbook of Meta-analysis in Ecology and Evolution.

[36] Johnson DW, Curtis PS (2001). Effects of forest management on soil C and N storage: Meta analysis. For. Ecol. Manage..

[37] Cardinale BJ (2006). Effects of biodiversity on the functioning of trophic groups and ecosystems. Nature.

[38] Chaudhary A, Burivalova Z, Koh LP, Hellweg S (2016). Impact of Forest Management on Species Richness: Global Meta-Analysis and Economic Trade-Offs. Sci. Rep..

[39] Oduor M (2016). Quantification of biochar effects on soil hydrological properties using meta-analysis of literature data. Geoderma.

[40] Steinfeld, H. *Livestock’s long shadow: environmental issues and opti-ons*. (2006).

[41] Manolikaki II, Mangolis A, Diamadopoulos E (2016). The impact of biochars prepared from agricultural residues on phosphorus release and availability in two fertile soils. J. Environ. Manage..

[42] Wu W (2012). Chemical characterization of rice straw-derived biochar for soil amendment. Biomass and Bioenergy.

[43] Guo, Y., Tang, H., Li, G. & Xie, D. Effects of cow dung biochar amendment on adsorption and leaching of nutrient from an acid yellow soil irrigated with biogas slurry. *Water. Air. Soil Pollut*. **225** (2014).

[44] Hass A (2012). Chicken Manure Biochar as Liming and Nutrient Source for Acid Appalachian Soil. J. Environ. Qual..

[45] Laird D, Fleming P, Wang B, Horton R, Karlen D (2010). Biochar impact on nutrient leaching from a Midwestern agricultural soil. Geoderma.

[46] Baronti Silvia, Alberti Giorgio, Delle Vedove Gemini, Di Gennaro Filippo, Fellet Guido, Genesio Lorenzo, Miglietta Franco, Peressotti Alessandro, Vaccari Francesco Primo (2010). The Biochar Option to Improve Plant Yields: First Results From Some Field and Pot Experiments in Italy. Italian Journal of Agronomy.

[47] Rondon MA, Lehmann J, Ramirez J, Hurtado M (2007). Biological nitrogen fixation by common beans (Phaseolus vulgaris L.) increases with bio-char additions. Biol. Fertil. Soils.

[48] Mia S (2014). Biochar application rate affects biological nitrogen fixation in red clover conditional on potassium availability. Agric. Ecosyst. Environ..

[49] Zheng H (2013). Characteristics and nutrient values of biochars produced from giant reed at different temperatures. Bioresour. Technol..

[50] Zwetsloot MJ, Lehmann J, Solomon D (2015). Recycling slaughterhouse waste into fertilizer: How do pyrolysis temperature and biomass additions affect phosphorus availability and chemistry?. J. Sci. Food Agric..

[51] Chintala R (2014). Phosphorus sorption and availability from biochars and soil/biochar mixtures. Clean - Soil, Air, Water.

[52] Parvage MM, Ulén B, Eriksson J, Strock J, Kirchmann H (2013). Phosphorus availability in soils amended with wheat residue char. Biol. Fertil. Soils.

[53] Vandecasteele B, Sinicco T, D’Hose T, Vanden Nest T, Mondini C (2016). Biochar amendment before or after composting affects compost quality and N losses, but not P plant uptake. J. Environ. Manage..

[54] Zhang H (2016). Roles of biochar in improving phosphorus availability in soils: A phosphate adsorbent and a source of available phosphorus. Geoderma.

[55] Farrell M, Macdonald LM, Butler G, Chirino-Valle I, Condron LM (2014). Biochar and fertiliser applications influence phosphorus fractionation and wheat yield. Biol. Fertil. Soils.

[56] Yuan H (2013). Influence of temperature on product distribution and biochar properties by municipal sludge pyrolysis. J. Mater. Cycles Waste Manag..

[57] Biederman LA, Stanley Harpole W (2013). Biochar and its effects on plant productivity and nutrient cycling: A meta-analysis. GCB Bioenergy.

[58] Christel W, Bruun S, Magid J, Kwapinski W, Jensen LS (2016). Pig slurry acidification, separation technology and thermal conversion affect phosphorus availability in soil amended with the derived solid fractions, chars or ashes. Plant Soil.

[59] Borchard N (2014). Black carbon and soil properties at historical charcoal production sites in Germany. Geoderma.

[60] Agegnehu G, Bass AM, Nelson PN, Bird MI (2016). Benefits of biochar, compost and biochar-compost for soil quality, maize yield and greenhouse gas emissions in a tropical agricultural soil. Sci. Total Environ..

[61] Fischer Daniel, Glaser Bruno (2012). Synergisms between Compost and Biochar for Sustainable Soil Amelioration. Management of Organic Waste.

[62] Schulz H, Glaser B (2012). Effects of biochar compared to organic and inorganic fertilizers on soil quality and plant growth in a greenhouse experiment. J. Plant Nutr. Soil Sci..

[63] Liu J (2012). Short-term effect of biochar and compost on soil fertility and water status of a Dystric Cambisol in NE Germany under field conditions. J. Plant Nutr. Soil Sci..

[64] Alotaibi K, Schoenau J (2016). Application of Two Bioenergy Byproducts with Contrasting Carbon Availability to a Prairie Soil: Three-Year Crop Response and Changes in Soil Biological and Chemical Properties. Agronomy.

