# Supplementary material for: Biochar effects on phosphorus availability in agricultural soils: A meta-analysis
Source: Sci Rep. 2019 Jun 27;9:9338. doi: 10.1038/s41598-019-45693-z (PMC6597700; doi:10.1038/s41598-019-45693-z)
Supplement: Supplementary file 1 — Supplementary Dataset 1 [file 41598_2019_45693_MOESM1_ESM.docx]

**Biochar effects on phosphorus availability in agricultural soils: A meta-analysis**

Verena Lehr and Bruno Glaser

Martin Luther University Halle-Wittenberg, Institute of Agronomy and Nutritional Sciences, Soil Biogeochemistry, von-Seckendorff-Platz 3, 06120 Halle/Saale, Germany

Corresponding author: [bruno.glaser@landw.uni-halle.de](mailto:bruno.glaser@landw.uni-halle.de)

**Supplementary Table 1** Summary of the data obtained from 107 pairwise comparisons used in this meta-analysis. X_E_ represents the mean plant-available phosphorus content of the soil amended with biochar and X_C_ is the mean plant-available phosphorus content of the un-amended soil. R is the response ratio calculated by X_E_/X_C_.

| **Studies** | **Feedstock** | **Pyrolysis Temperature**  **[°C]** | **Amount**  **[Ma ha^-1^]** | **Soil pH** | **Time** | **Extraction**  **Method** | **X_E_**  **[mg/kg]** | **X_C_**  **[mg/kg]** | **ln(R)** | **R** |
| --- | --- | --- | --- | --- | --- | --- | --- | --- | --- | --- |
|  |  |  |  |  |  |  |  |  |  |  |
| Alotabi & Schoenau, 2016 | Oat hulls | 450 | 2.8 | 7.9 | 3 years | modified Kelowna extraction | 14.5 | 13.7 | 0.06 | 1.06 |
| Bai, Hosseini *et al.* 2014 | Poultry litter | 500-550 | 10 | n/a | 5 years | Bray No. 1 | 51.6 | 4.68 | 2.4 | 11.03 |
| Bai, Hosseini *et al*. 2014 | Green waste | 500-550 | 10 | n/a | 5 years | Bray No. 1 | 10 | 4.68 | 0.76 | 2.14 |
| Brantley *et al.* 2016 | Poultry litter | 500-520 | 5 | 6.5 | 92 days | Mehlich III | 1.1 | 1 | 0.1 | 1.10 |
| Brantley *et al.* 2016 | Poultry litter | 500-520 | 10 | 6.5 | 92 days | Mehlich III | 1.3 | 1 | 0.26 | 1.3 |
| Cavoski *et al.* 2016 | Olive-mill waste | 1100-1200 | 5 | 7.5 | 16 weeks | Olsen | 7 | 7 | 0 | 1.00 |
| Chathurika *et al*. 2016 | Wood chip | 500-650 | 30 | 8.0 | 70 days | Olsen | 6.8 | 7.6 | -0.11 | 0.89 |
| Chathurika *et al*. 2016 | Wood chip | 500-650 | 30 | 7.6 | 70 days | Olsen | 6.8 | 7.8 | -0.14 | 0.87 |
| Chathurika *et al*. 2016 | Wood chip | 500-650 | 15 | 8.0 | 70 days | Olsen | 7 | 8.2 | -0.16 | 0.85 |
| Chathurika *et al*. 2016 | Wood chip | 500-650 | 15 | 7.6 | 70 days | Olsen | 7 | 8.7 | -0.22 | 0.80 |
| Dai *et al.* 2013 | Reed | 500 | 20 | 4.7 | 100 days | Olsen | 37.51 | 33.73 | 0.11 | 1.11 |
| Dai *et al.* 2013 | Reed | 500 | 60 | 4.7 | 100 days | Olsen | 52.38 | 33.73 | 0.44 | 1.55 |
| **Studies** | **Feedstock** | **Pyrolysis Temperature**  **[°C]** | **Amount**  **[Ma ha^-1^]** | **Soil pH** | **Time** | **Extraction**  **Method** | **X_E_**  **[mg/kg]** | **X_C_**  **[mg/kg]** | **ln(R)** | **R** |
|  |  |  |  |  |  |  |  |  |  |  |
| Dai *et al.* 2013 | Pig manure | 500 | 20 | 4.7 | 100 days | Olsen | 129.41 | 33.73 | 1.34 | 3.84 |
| Dai *et al.* 2013 | Pig manure | 500 | 60 | 4.7 | 100 days | Olsen | 175.2 | 33.73 | 1.65 | 5.19 |
| Dai *et al.* 2013 | Pineapple Peel | 500 | 20 | 4.7 | 100 days | Olsen | 62.28 | 33.73 | 0.61 | 1.85 |
| Dai *et al.* 2013 | Pineapple Peel | 500 | 60 | 4.7 | 100 days | Olsen | 115.93 | 33.73 | 1.23 | 3.44 |
| Gao *et al*. 2016 | Logging residue | 500 | 20 | 5.9 | 4 months | CaCl_2_ | 10.99 | 10.34 | 0.06 | 1.06 |
| Hossain *et al.* 2010 | Wastewater sludge | 550 | 10 | 4.6 | 16 weeks | Colwell | 56 | 26 | 0.77 | 2.15 |
| Hunt *et al*. 2013 | Dairy manure | 350 | 5 | 4.5 | 51 days | modified Mehlich III | 25.4 | 10.3 | 0.9 | 2.47 |
| Hunt *et al*. 2013 | Dairy manure | 700 | 2.5 | 4.5 | 51 days | modified Mehlich III | 23.4 | 10.3 | 0.82 | 2.27 |
| Hunt *et al*. 2013 | Beef manure | 350 | 4 | 4.5 | 51 days | modified Mehlich III | 23.2 | 10.3 | 0.81 | 2.25 |
| Hunt *et al*. 2013 | Beef manure | 700 | 2.5 | 4.5 | 51 days | modified Mehlich III | 20.7 | 10.3 | 0.7 | 2.01 |
| Hunt *et al*. 2013 | Chicken manure | 350 | 2 | 4.5 | 51 days | modified Mehlich III | 21.8 | 10.3 | 0.75 | 2.12 |
| Hunt *et al*. 2013 | Chicken manure | 700 | 1.5 | 4.5 | 51 days | modified Mehlich III | 19.9 | 10.3 | 0.66 | 1.93 |
| Hunt *et al*. 2013 | Turkey manure | 350 | 1.5 | 4.5 | 51 days | modified Mehlich III | 20.5 | 10.3 | 0.69 | 1.99 |
| **Studies** | **Feedstock** | **Pyrolysis Temperature**  **[°C]** | **Amount**  **[Ma ha^-1^]** | **Soil pH** | **Time** | **Extraction**  **Method** | **X_E_**  **[mg/kg]** | **X_C_**  **[mg/kg]** | **ln(R)** | **R** |
|  |  |  |  |  |  |  |  |  |  |  |
| Hunt *et al*. 2013 | Turkey manure | 700 | 1.5 | 4.5 | 51 days | modified Mehlich III | 19.7 | 10.3 | 0.65 | 1.91 |
| Hunt *et al*. 2013 | Pig manure | 350 | 1.5 | 4.5 | 51 days | modified Mehlich III | 19.6 | 10.3 | 0.64 | 1.90 |
| Hunt *et al*. 2013 | Pig manure | 700 | 1 | 4.5 | 51 days | modified Mehlich III | 20.7 | 10.3 | 0.70 | 2.01 |
| Jin *et al*. 2016 | Pig manure | 400 | 10 | 6.3 | 98 days | Olsen | 87.2 | 20.9 | 1.43 | 4.17 |
| Jin *et al*. 2016 | Pig manure | 400 | 30 | 6.3 | 98 days | Olsen | 141.6 | 20.9 | 1.91 | 6.78 |
| Jin *et al.* 2016 | Pig manure | 400 | 10 | 5.0 | 98 days | Olsen | 44.5 | 12.2 | 1.29 | 3.65 |
| Jin *et al.* 2016 | Pig manure | 400 | 30 | 5.0 | 98 days | Olsen | 109.6 | 12.2 | 2.20 | 8.98 |
| Marchetti & Castelli 2013 | Wood chip | 420 | 10 | 8.2 | 90 days | Olsen | 26 | 26 | 0 | 1.00 |
| Marchetti & Castelli 2013 | Swine solids | 420 | 10 | 8.2 | 90 days | Olsen | 60.1 | 26 | 0.84 | 2.31 |
| Naggar et al.  2015 | Conocarpus wood waste | 400 | 20 | 8.5 | 90 days | AB-DTPA | 0.57 | 0.32 | 0.58 | 1.78 |
| Novak *et al.*  2009 | Peanut hull | 400 | 40 | 5.9 | 2 days | Mehlich I | 104 | 5.9 | 2.87 | 17.63 |
| Novak *et al*.  2009 | Peanut hull | 500 | 40 | 5.9 | 2 days | Mehlich I | 85 | 5.9 | 2.67 | 14.41 |
| Novak *et al.*  2009 | Pecan shell | 350 | 40 | 5.9 | 2 days | Mehlich I | 71 | 5.9 | 2.49 | 12.03 |
| **Studies** | **Feedstock** | **Pyrolysis Temperature**  **[°C]** | **Amount**  **[Ma ha^-1^]** | **Soil pH** | **Time** | **Extraction**  **Method** | **X_E_**  **[mg/kg]** | **X_C_**  **[mg/kg]** | **ln(R)** | **R** |
|  |  |  |  |  |  |  |  |  |  |  |
| Novak *et al.*  2009 | Pecan shell | 700 | 40 | 5.9 | 2 days | Mehlich I | 71 | 5.9 | 2.49 | 12.03 |
| Novak *et al.*  2009 | Switch grass | 250 | 40 | 5.9 | 2 days | Mehlich I | 74 | 5.9 | 2.53 | 12.54 |
| Novak *et al.*  2009 | Switch grass | 500 | 40 | 5.6 | 2 days | Mehlich I | 94 | 5.9 | 2.77 | 15.93 |
| Novak & Buscher 2013 | Peanut hull | 400 | 40 | 5.6 | 120 days | Mehlich I | 39 | 29 | 0.30 | 1.34 |
| Novak & Buscher 2013 | Peanut hull | 500 | 40 | 5.6 | 120 days | Mehlich I | 33 | 29 | 0.13 | 1.14 |
| Novak & Buscher 2013 | Hard wood | 700 | 40 | 5.6 | 120 days | Mehlich I | 22 | 29 | -0.28 | 0.76 |
| Novak *et al.* 2014 | Peanut hull | 400 | 40 | 5.6 | 127 days | Mehlich I | 36 | 27 | 0.29 | 1.33 |
| Novak *et al.* 2014 | Peanut hull | 500 | 40 | 5.6 | 127 days | Mehlich I | 28 | 27 | 0.04 | 1.04 |
| Novak *et al.* 2014 | Pecan shell | 350 | 40 | 5.6 | 127 days | Mehlich I | 24 | 27 | -0.12 | 0.89 |
| Novak *et al.* 2014 | Pecan shell | 700 | 40 | 5.6 | 127 days | Mehlich I | 31 | 27 | 0.14 | 1.15 |
| Novak *et al.* 2014 | Poultry litter | 350 | 40 | 5.6 | 127 days | Mehlich I | 393 | 27 | 2.68 | 14.56 |
| Novak *et al.* 2014 | Poultry litter | 700 | 40 | 5.6 | 127 days | Mehlich I | 714 | 27 | 3.28 | 26.44 |
| Novak *et al.* 2014 | Switch grass | 250 | 40 | 5.6 | 127 days | Mehlich I | 29 | 27 | 0.07 | 1.07 |
| **Studies** | **Feedstock** | **Pyrolysis Temperature**  **[°C]** | **Amount**  **[Ma ha^-1^]** | **Soil pH** | **Time** | **Extraction**  **Method** | **X_E_**  **[mg/kg]** | **X_C_**  **[mg/kg]** | **ln(R)** | **R** |
|  |  |  |  |  |  |  |  |  |  |  |
| Novak *et al.* 2014 | Hard wood waste | 500 | 40 | 5.6 | 127 days | Mehlich I | 22 | 27 | -0.2 | 0.81 |
| Novak *et al.* 2015 | Pig solids | 350 | 10 | 6.5 | 124 days | Mehlich I | 155 | 21 | 2.00 | 7.38 |
| Novak *et al.* 2015 | Pig solids | 350 | 20 | 6.5 | 124 days | Mehlich I | 212 | 21 | 2.31 | 10.10 |
| Novak *et al.* 2015 | Pig solids | 350 | 40 | 6.5 | 124 days | Mehlich I | 490 | 21 | 3.15 | 23.33 |
| Olmo *et al*. 2014 | Olive-tree pruning | 450 | 40 | 8.2 | 7 months | Olsen | 21 | 12.8 | 0.5 | 1.64 |
| Partey *et al*. 2014 | Mixed hardwood | 500 | 5 | 6.1 | 1 year | Olsen | 5.6 | 5.4 | 0.04 | 1.04 |
| Parvage *et al.*  2013 | Wheat residue | 500 | 20 | 6.4 | 16 hours | WSP | 4.43 | 2.76 | 0.47 | 1.61 |
| Parvage *et al.*  2013 | Wheat residue | 500 | 20 | 6 | 16 hours | WSP | 1.55 | 1.11 | 0.33 | 1.40 |
| Parvage *et al.*  2013 | Wheat residue | 500 | 20 | 6.2 | 16 hours | WSP | 0.95 | 0.66 | 0.36 | 1.44 |
| Parvage *et al.*  2013 | Wheat residue | 500 | 20 | 6.6 | 16 hours | WSP | 5.25 | 4.74 | 0.10 | 1.11 |
| Parvage *et al.*  2013 | Wheat residue | 500 | 20 | 5.4 | 16 hours | WSP | 7.61 | 3.96 | 0.65 | 1.92 |
| Parvage *et al.*  2013 | Wheat residue | 500 | 20 | 7.7 | 16 hours | WSP | 0.53 | 0.15 | 1.26 | 3.53 |
| Parvage *et al.*  2013 | Wheat residue | 500 | 20 | 6.2 | 16 hours | WSP | 1.14 | 1.2 | -0.05 | 0.95 |
| **Studies** | **Feedstock** | **Pyrolysis Temperature**  **[°C]** | **Amount**  **[Ma ha^-1^]** | **Soil pH** | **Time** | **Extraction**  **Method** | **X_E_**  **[mg/kg]** | **X_C_**  **[mg/kg]** | **ln(R)** | **R** |
|  |  |  |  |  |  |  |  |  |  |  |
| Parvage *et al.*  2013 | Wheat residue | 500 | 20 | 5.9 | 16 hours | WSP | 7.23 | 6.03 | 0.18 | 1.20 |
| Parvage *et al.*  2013 | Wheat residue | 500 | 20 | 5.9 | 16 hours | WSP | 0.09 | 0.06 | 0.41 | 1.50 |
| Parvage *et al.*  2013 | Wheat residue | 500 | 20 | 5.3 | 16 hours | WSP | 1.17 | 0.63 | 0.62 | 1.86 |
| Parvage *et al*.  2013 | Wheat residue | 500 | 20 | 5.3 | 16 hours | WSP | 0.12 | 0.12 | 0 | 1 |
| Warren *et al*.  2009 | Cattle bone | 400 | 7.6 | 7.9 | 145 days | Olsen | 44.7 | 56.6 | -0.04 | 0.96 |
| Warren *et al*.  2009 | Cattle bone | 400 | 7.6 | 6.1 | 145 days | Olsen | 68.7 | 50.2 | 0.31 | 1.37 |
| Warren *et al*.  2009 | Cattle bone | 400 | 7.6 | 6.8 | 145 days | Olsen | 41.2 | 39.2 | 0.05 | 1.05 |
| Warren *et al*.  2009 | Cattle bone | 400 | 7.6 | 8.3 | 145 days | Olsen | 14.9 | 13.4 | 0.11 | 1.11 |
| Warren *et al*.  2009 | Cattle bone | 400 | 7.6 | 7.4 | 145 days | Olsen | 25.6 | 13.9 | 0.61 | 1.84 |
| Warren *et al*.  2009 | Cattle bone | 400 | 7.6 | 5.0 | 145 days | Olsen | 70.8 | 5.3 | 2.59 | 13.36 |
| Warren *et al*.  2009 | Cattle bone | 400 | 7.6 | 5.5 | 145 days | Olsen | 127.8 | 67.7 | 0.64 | 1.89 |
| Warren *et al*.  2009 | Cattle bone | 400 | 7.6 | 5.0 | 145 days | Olsen | 65 | 1.4 | 3.84 | 46.43 |
| Warren *et al*.  2009 | Cattle bone | 400 | 7.6 | 5.4 | 145 days | Olsen | 47.1 | 12.1 | 1.36 | 3.89 |
| **Studies** | **Feedstock** | **Pyrolysis Temperature**  **[°C]** | **Amount**  **[Ma ha^-1^]** | **Soil pH** | **Time** | **Extraction**  **Method** | **X_E_**  **[mg/kg]** | **X_C_**  **[mg/kg]** | **ln(R)** | **R** |
|  |  |  |  |  |  |  |  |  |  |  |
| Warren *et al*.  2009 | Cattle bone | 400 | 7.6 | 5.1 | 145 days | Olsen | 44 | 12.8 | 1.23 | 3.44 |
| Warren *et al*.  2009 | Cattle bone | 400 | 7.6 | 8.8 | 145 days | Olsen | 13.1 | 11 | 0.17 | 1.19 |
| Warren *et al*.  2009 | Cattle bone | 400 | 7.6 | 3.4 | 145 days | Olsen | 146.7 | 11.2 | 2.57 | 13.10 |
| Wu *et al*. 2014 | Furfural residue | 300 | 4.5 | 8.3 | 56 days | Olsen | 7.43 | 3.34 | 0.80 | 2.22 |
| Wu *et al*. 2014 | Furfural residue | 300 | 4.5 | 8.3 | 56 days | Olsen | 12.42 | 3.34 | 1.31 | 3.72 |
| Xu *et al.* 2015 | Peanut shell | 550 | 9.2 | 5.5 | 163 days | Olsen | 3 | 4.8 | -0.47 | 0.63 |
| Xu *et al.* 2015 | Peanut shell | 550 | 9.2 | 5.5 | 163 days | Olsen | 7.2 | 6.1 | 0.17 | 1.18 |
| Zhai *et al.* 2015 | Maize straw | 400 | 40 | 6.4 | 42 days | Olsen | 12 | 3 | 1.39 | 4.00 |
| Zhai *et al.* 2015 | Maize straw | 400 | 75 | 6.4 | 42 days | Olsen | 27 | 3 | 2.20 | 9.00 |
| Zhai *et al.* 2015 | Maize straw | 400 | 150 | 6.4 | 42 days | Olsen | 46 | 3 | 2.73 | 15.33 |
| Zhai *et al.* 2015 | Maize straw | 400 | 40 | 8.3 | 42 days | Olsen | 53 | 13 | 1.41 | 4.08 |
| Zhai *et al.* 2015 | Maize straw | 400 | 75 | 8.3 | 42 days | Olsen | 93 | 13 | 1.97 | 7.15 |
| Zhai *et al.* 2015 | Maize straw | 400 | 150 | 8.3 | 42 days | Olsen | 137 | 13 | 2.36 | 10.54 |
| **Studies** | **Feedstock** | **Pyrolysis Temperature**  **[°C]** | **Amount**  **[Ma ha^-1^]** | **Soil pH** | **Time** | **Extraction**  **Method** | **X_E_**  **[mg/kg]** | **X_C_**  **[mg/kg]** | **ln(R)** | **R** |
|  |  |  |  |  |  |  |  |  |  |  |
| Zhao *et al.* 2014 | Maize straw | 500 | 10 | 8.6 | 14 days | Olsen | 10.96 | 10 | 0.09 | 1.10 |
| Zhao *et al.* 2014 | Maize straw | 500 | 20 | 8.6 | 14 days | Olsen | 13.2 | 10 | 0.28 | 1.32 |
| Zhao *et al.* 2014 | Maize straw | 500 | 40 | 8.6 | 14 days | Olsen | 15.46 | 10 | 0.44 | 1.55 |
| Zhao *et al.* 2014 | Maize straw | 500 | 95 | 8.6 | 14 days | Olsen | 25.36 | 10 | 0.93 | 2.54 |
| Zhao *et al.* 2014 | Maize straw | 500 | 10 | 5.3 | 14 days | Olsen | 6.94 | 6.02 | 0.14 | 1.15 |
| Zhao *et al.* 2014 | Maize straw | 500 | 20 | 5.3 | 14 days | Olsen | 8.16 | 6.02 | 0.30 | 1.36 |
| Zhao *et al.* 2014 | Maize straw | 500 | 40 | 5.3 | 14 days | Olsen | 7.91 | 6.02 | 0.27 | 1.31 |
| Zhao *et al.* 2014 | Maize straw | 500 | 95 | 5.3 | 14 days | Olsen | 12.7 | 6.02 | 0.75 | 2.11 |
| Zong *et al*. 2016 | Wheat straw | 500 | 40 | 5.0 | 180 days | Olsen | 20.37 | 7.18 | 1.04 | 2.84 |
| Zong *et al*. 2016 | Wheat straw | 500 | 75 | 5.0 | 180 days | Olsen | 43.5 | 7.18 | 1.80 | 6.06 |
| Zong *et al*. 2016 | Wheat straw | 500 | 115 | 5.0 | 180 days | Olsen | 63.24 | 7.18 | 2.18 | 8.81 |
| Zong *et al*. 2016 | Wood chips | 500 | 40 | 5.0 | 180 days | Olsen | 14.26 | 7.18 | 0.69 | 1.99 |
| Zong *et al*. 2016 | Wood chips | 500 | 75 | 5.3 | 180 days | Olsen | 18.05 | 7.18 | 0.92 | 2.51 |
| **Studies** | **Feedstock** | **Pyrolysis Temperature**  **[°C]** | **Amount**  **[Ma ha^-1^]** | **Soil pH** | **Time** | **Extraction**  **Method** | **X_E_**  **[mg/kg]** | **X_C_**  **[mg/kg]** | **ln(R)** | **R** |
|  |  |  |  |  |  |  |  |  |  |  |
| Zong *et al*. 2016 | Wood chips | 500 | 115 | 5.0 | 180 days | Olsen | 21.12 | 7.18 | 1.08 | 2.94 |
| Zong *et al*. 2016 | Wastewater sludge | 500 | 40 | 5.0 | 180 days | Olsen | 23.5 | 7.18 | 1.19 | 3.27 |
| Zong *et al*. 2016 | Wastewater sludge | 500 | 75 | 5.0 | 180 days | Olsen | 55.11 | 7.18 | 2.04 | 7.68 |
| Zong *et al*. 2016 | Wastewater sludge | 500 | 115 | 5.0 | 180 days | Olsen | 122.5 | 7.18 | 2.84 | 17.06 |
